# Reimagined MPFL Reconstruction: Retinacular Fixation of the Doubled Hamstring Graft at the Patella and Suture Anchor-Based Femoral Fixation

**DOI:** 10.1155/2023/6647760

**Published:** 2023-09-14

**Authors:** Muhammed Ehsan Nazeer, Sagar Goel, Muhammed Nazeer, Gowrishankar Sreenivasan, Mohsin Nazeer Muhammed, Suzaan Shajil

**Affiliations:** ^1^Orthopedics, Cumberland Infirmary, North Cumbria Integrated Care Trust, Carlisle, UK; ^2^Department of Orthopedics, KIMS Health, Thiruvananthapuram, Kerala, India; ^3^Milton Keynes Hospital, Milton Keynes, UK; ^4^NHS Lothian, Royal Infirmary, Edinburgh, UK

## Abstract

**Background:**

Lateral patellar dislocation is frequently observed among teenagers and young adults. There is no consensus on the best type of graft or fixation strategy for the femur and patella, and complications such as iatrogenic patella fracture, tunnel malposition, and grafting failure are common. The objective of our research is to find out the functional outcome of a new method of medial patellofemoral ligament (MPFL) reconstruction, which involves two key components: (1) patellar fixation is accomplished by suturing the two limbs of the looped doubled hamstring graft in a divergent fashion to the retinaculum at the medial border of the upper half of patella and (2) the placement of a suture anchor tied to the graft at the isometric point on the medial femur condyle.

**Methods:**

This study is a retrospective assessment of patients who underwent MPFL reconstruction at our hospital between September 2018 and August 2020. Patients were monitored for at least 2 years after the initial procedure until August 2022.

**Results:**

A total of 29 patients were recruited for the study, with 22 being females and the average age being 30.38 years. During the postoperative period, none of the participants experienced instability, redislocation, patellar/femoral fractures, or abnormal distal femur growth. The Tegner–Lysholm knee score was good to excellent for 17 (58.6%) participants, fair for 10 (34.5%) participants, and poor for 2 (6.9%) participants. The Kujala anterior knee pain score was more than 80 for 19 (65.5%) participants.

**Conclusion:**

This research presents a significant achievement rate of the surgical procedure, accompanied by the mean Tegner–Lysholm knee score of 82.68 and the mean Kujala anterior knee pain score of 82.71. Notably, there were no complications observed in the postoperative period.

## 1. Introduction

Lateral patellar dislocation is frequently observed among teenagers and young adults. While many doctors prescribe rest and rehabilitation as a standard treatment for initial patellar dislocations [1, 2], research suggests that nonoperative treatments have varying outcomes with a recurrence rate ranging from 15% to 44% [3]. In persons who have had two prior episodes of dislocation, the recurrence rates jump to 49% [4]. The medial patellofemoral ligament (MPFL) is widely regarded as the most important medial stabilizing structure for the patella, preventing subluxation and dislocation [5, 6]. Therefore, MPFL reconstruction is the most commonly performed surgical procedure as it is found to be injured in 90% of people with acute lateral patellar dislocation [7, 8]. Various techniques for MPFL reconstruction have been described in the past, but no single technique has been established as the standard or preferred method. In addition, there is no consensus on the best type of graft or fixation strategies for the femur and patella, and complications such as iatrogenic patellar fracture, femoral tunnel malposition, and graft failure are common. Patellar fixation techniques include single or double transosseous tunnels (either transverse from medial to lateral or oblique from medial to anterior or blind tunnels confined to medial one third of patella) or fixing the graft on the surface of patella using suture anchors. However, all these techniques can create a weak spot on the patella, making it vulnerable to fractures when subjected to direct or indirect forces. Poor tunnel placement and the use of large-sized tunnels can also lead to patellar fractures [9]. Dhinsa et al. [5] reported two cases of patellar fracture after MPFL reconstruction where patellar fixation was performed using suture anchors. Mikashima et al. [10] reported two patellar fractures in a group of 12 patients when the graft was passed through a 4.5-mm patellar bone tunnel. A review [11] of multiple studies found that the incidence of patellar fracture is not high enough to draw any clear conclusions but suggested using a technique that carries less risk of fracture, such as a docking anchor or suture fixation.

The femoral fixation techniques for MPFL reconstruction involve passing the graft through a tunnel in the femur. However, in immature skeletons, this poses a risk of injury to the physis due to the tunnel's proximity to the distal femoral epiphysis. Although this complication is rare, it has been reported in the literature. For instance, Arianna Trionfo, Ajay Shah, and others reported the first case of lateral physeal growth arrest and subsequent coronal plane deformity following MPFL reconstruction in a skeletally immature patient [12]. Gerd Seitlinger and colleagues also reported a case of distal femoral physeal injury during femoral tunnel placement of an anatomic MPFL reconstruction in a skeletally immature patient [13]. A cadaveric study by Nguyen emphasized the importance of safe drilling paths in the distal femoral epiphysis for pediatric MPFL reconstruction to prevent injury to the distal femoral physis [14].

The objective of our research is to find out the functional outcome of a new method of MPFL reconstruction, which involves two key components: (1) patellar fixation is accomplished by suturing the two limbs of the looped doubled hamstring graft in a divergent fashion to the retinaculum at the medial border of the upper half of patella and (2) the placement of a suture anchor tied to the graft at the isometric point on the medial femur condyle.

## 2. Materials and Methods

The study, which was conducted between September 2020 and August 2022, is a retrospective assessment of patients who underwent MPFL reconstruction in our hospital between September 2018 and August 2020. Before the study began, institutional ethics approval was obtained. The hospital's electronic medical database was examined to identify patients who had undergone MPFL reconstruction for an isolated tear in the MPFL as seen in MRI scans. Patients aged 50 or above, those with simultaneous multiligament injury, or having fractures around the knee were excluded. The criteria for inclusion did not define a lower age limit. Patients with lateralized tuberosity, hypoplastic lateral femoral condyle or high-grade trochlear dysplasia, and those who underwent concurrent procedures such as lateral retinaculum release or osteotomy were excluded from the study. From September 2018 to August 2020, a total of 37 patients underwent MPFL reconstruction. Among them, 3 had simultaneous anterior cruciate ligament reconstruction, 3 had additional lateral retinaculum release, and 1 patient had a tibial tuberosity osteotomy simultaneously. While 30 patients met the criteria of inclusion and exclusion, one patient was excluded due to loss of follow-up. All 29 participants were informed of the objectives, goals, and methods of the study and obtained informed consent. Patients were evaluated for patellar instability and redislocation for a minimum of two years after the reconstruction of the MPFL. The functional rating was based on the Tegner–Lysholm knee score and Kujala score.

### 2.1. Surgical Approach

**Figure 1:**
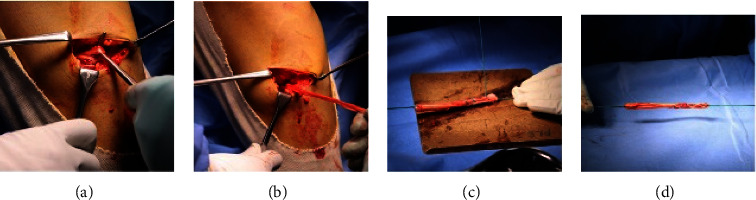
The semitendinosus tendon is located and obtained using a closed tendon stripper, as shown in (a, b). The ends of the tendon are secured with a nonabsorbable suture (size 5 ethibond) as seen in (c, d). The tendon is then folded to achieve a minimum diameter of 7 mm and length of 10 cm. Graft is pretensioned using a graft tensioning board with a maximum one hand pull.

With the patient under appropriate anaesthesia and a single dose of prophylactic parental antibiotic, the patient is positioned supine with the ipsilateral knee and leg hanging down from the thigh post, with a tourniquet in place, and the contralateral thigh held in thigh abduction post. The semitendinosus graft is harvested and prepared under tourniquet control using an obliquely placed 5-6 cm linear incision medial to the tibial tuberosity. The pes anserinus and the semitendinosus tendon are identified and harvested using a closed tendon stripper. The ends of the tendon are secured using size 5 Ethibond (nonabsorbable suture), and the tendon is doubled to achieve a minimum diameter of 7 mm and length of 10 cm (Figure 1).

**Figure 2:**
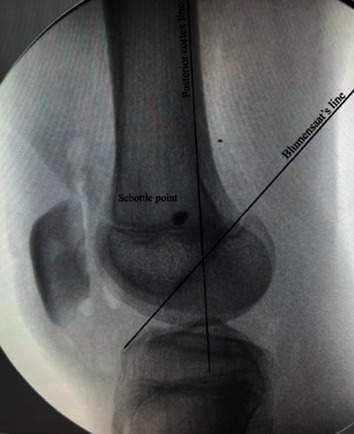
Shuttle point, posterior cortex line, and Blumensaat's line.

To expose the medial femoral epicondyle, the knee is flexed to 90 degrees and the Schottle point, as described by Schottle et al. [15], is located using a 2 mm K-wire under an image intensifier. A metallic, cock screw type suture anchor with attached fibre wires of size 5 mm × 15.5 mm is then introduced at the same location, with the K-wire serving as a guide (Figure 2).

**Figure 3:**
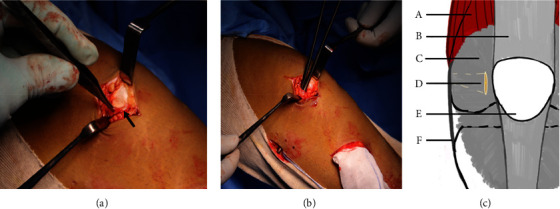
(a) The black arrow indicates the extensor retinaculum. (b) Artery forceps are inserted between the extensor retinaculum and joint capsule to indicate the soft tissue tunnel. (c) The quadriceps muscle is labeled as (A), the quadriceps tendon as (B), the extensor retinaculum as (C), the soft tissue tunnel as (D), the patella as (E), and the medial collateral ligament as (F).

An incision of 4-5 cm is made along the medial border of the patella. The prepatellar tissue, which is layer 1, is lifted up from the medial border of the patella, and the retinaculum, which is layer 2, is exposed. Using a tendon tunneler, a soft tissue tunnel is created between the medial patellar retinaculum (layer 2) and joint capsule (layer 3) up to the Schottle point through sharp dissection (Figure 3).

**Figure 4:**
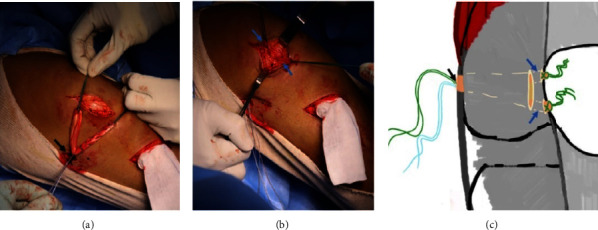
(a) The point where the double graft is secured at the middle to the suture anchor on the medial femoral condyle is indicated by the black arrow. (b) The two limbs of the graft are pulled through the soft tissue tunnel towards the medial border of the patella, as shown by the blue arrows. (c) The medial part of the graft is secured at the Schottle point, as indicated by the black arrow. The ends of the two limbs of the graft are embedded in the retinaculum at the medial border of the patella, as shown by the two blue arrows.

A passage is created between the second layer (retinaculum) and the third layer (joint capsule) without causing damage to the capsule or synovium of the knee joint. The double graft is tied to the suture anchor at the midpoint in order to achieve equal limbs and is looped at the Schottle point to enable its two limbs to reach the medial border of the patella. The two limbs of the graft are then passed divergently through the soft tissue tunnel to reach the upper half of the medial border of the patella (See Figure 4). Tension on the graft is applied through a manual pull on the patellar side keeping the knee at 45 degrees of flexion.

**Figure 5:**
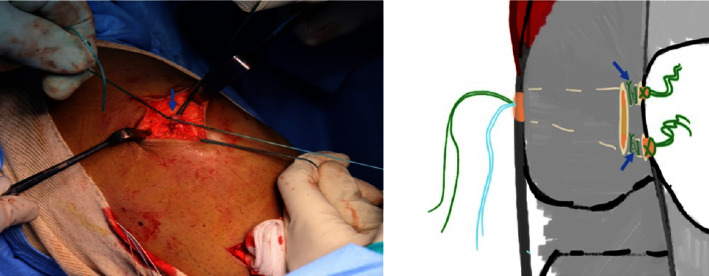
The blue arrows represent the fixation of the graft's limbs with the retinaculum located at the medial border of the patella using Ethibond.

Once the appropriate tension of the graft at 45-degree knee flexion is confirmed, a suture bite using Ethibond is taken through the retinaculum, one limb of the graft, and then through the retinaculum again. This process is repeated for the other limb of the graft, which is secured to the retinaculum in a divergent manner (Figure 5).

**Figure 6:**
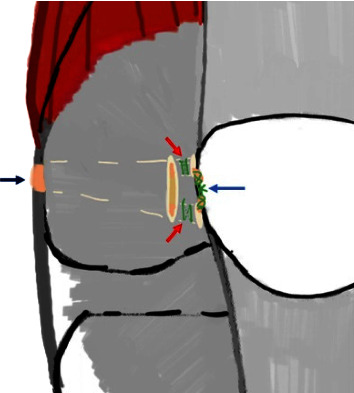
The point where the medial end of the graft is secured is indicated by the black arrow, while the retinacula at the medial border of the patella, represented by the red arrows, fix the two limbs of the graft. The two limbs of the graft are also tied with each other to provide additional stability and strength to the fixation, as indicated by the blue arrow.

To increase the stability of the fixation, the two limbs of the graft are tied together using the Ethibond that was previously tied to their ends (Figure 6).

Intraoperatively, the tension of the graft, patellar shift test, and patellar tracking are assessed and are followed by a wound closure. The patient is then given a sterile dressing and compression bandage, followed by a knee brace in full extension to maintain the knee's position. After the surgery, the knee is kept in full extension for the first two weeks, and isometric quadriceps and hamstring exercises are started. The patient is allowed to bear weight cautiously, and short arc range of motion exercises are permitted starting from the second week, with a long arc range of motion exercises beginning in the fifth week. The ROM knee brace is removed after six weeks, and the patient is encouraged to do full range of motion exercises. The patient can resume sports activities after six months.

## 3. Results

The study enrolled 29 participants, with 22 being females and the average age being 30.38. The youngest participant was 17 years old, while the oldest was 49 years old. During the postoperative period, none of the participants reported instability, redislocation, or patellar/femoral fractures. The mean lateral patellar translation and patellar tracking were normal, with a negative apprehension test for all participants. The Tegner–Lysholm knee score was good to be excellent for 17 (58.6%) participants, fair for 10 (34.5%) participants, and poor for 2 (6.9%) participants. The Kujala anterior knee pain score was more than 80 for 19 (65.5%) participants, and none of the participants scored less than 60 on the Kujala anterior knee pain scale. The mean Tegner–Lysholm knee score and Kujala anterior knee pain score were 82.68 and 82.71, respectively.

## 4. Discussion

Nonoperative management of MPFL tear has reported a recurrence rate of 15%–44% in patients with one episode of lateral patellar dislocation [3] and 49% in patients with two episodes of dislocation [4]. This study does not report any case of instability and redislocation in the postoperative period. In a cadaveric knee study, Laprade RF, Anders Hauge Engebresten et al. [16] found that the MPFL is located just in front of the deeper medial joint capsule, which is consistent with Warren and Marshall's [17] three-layered system concept. According to this system, the MPFL is part of layer 2, which comprises transverse fibers that pass between the medial femur epicondyle and patella, and it blends with the medial retinaculum near the patellar insertion [17, 18]. In the present surgical technique, we made a soft tissue tunnel between layers 2 and 3 to maintain the extracapsular nature of the donor MPFL graft anterior to the deeper joint capsule. We selected the Schottle point as the femoral attachment point of the suture anchor, as described by Schottle [15]. In skeletally immature patients, the anatomical point used for femoral attachment was carefully identified to avoid any damage to the distal femoral growth plate. Out of the three patients with open growth plates, none of the patients showed any clinical or radiological signs of physeal injury. However, Trionfo et al. reported the first case of lateral growth arrest and subsequent coronal plane deformity after MPFL reconstruction in a young patient [12], and Seitlinger et al. also reported a case of physeal injury during femoral tunnel placement in a skeletally immature patient [13]. Farrow and colleagues, in their cadaveric study, found that the anatomic MPFL attachment site is very close to the distal femoral growth plate [19]. Liu et al. conducted a study on the structure and position of the distal femoral physis and stressed that it is a complex structure that is at a risk of damage during the drilling of the femoral tunnel, even if the tunnel does not breach the physis [20]. The authors noted that drilling too close to the physis could result in thermal damage. Similarly, Nguyen's cadaveric study highlighted the significance of safe drilling paths in the distal femoral epiphysis during pediatric MPFL reconstruction to prevent injury to the physis [14]. Here, we have used a suture anchor instead of creating bone tunnels for femoral fixation of the graft which mitigated the inherent risks associated with femoral tunnels as found in the literature.

Several methods have been described for fixing the patella during MPFL reconstruction, including the use of single, double, or incomplete bone tunnels, interference screws, and suture anchor fixations. Incorrect tunnel placement or the use of large-sized tunnels can increase the risk of iatrogenic patellar fractures [9], as reported by Mikashima et al. [10]. Reaming tunnels may also damage the articular cartilage, leading to postoperative anterior knee pain and early-onset patellofemoral arthritis. To minimize the risk of violating the articular cartilage, Makovicka et al. [21] advocated for the use of suture anchors. However, each fixation technique causes a stress riser in the patella that can lead to patellar fractures under direct or indirect forces, as reported by Dhinsa et al. [22] who reported two cases of patellar fractures after MPFL reconstruction using suture anchors. Shah's systemic review [11] did not find a conclusive incidence of patellar fracture but recommended considering a technique that does not carry the risk of fracture such as a docking anchor or suture fixation. In our surgical approach, we utilized soft tissue suture fixation for patellar fixation. The two limbs of the graft were fixed in a divergent fashion to the medial retinaculum at the medial border of superior half patella. This was performed to maintain an adequate and efficient anatomical pull to the patella medially. The two limbs of the graft created an equal vector of medial force on the upper half of the patella. A meta-analysis has found a high combined complication and failure rate of MPFL reconstruction, with patellar fracture, articular damage, and distal femoral physeal damage being a small proportion of all complications but still significant due to their impact on patients' quality of life. The researchers categorically divided the surgical methods into two main groups: tunnel fixation and suture fixation. Participants who underwent tunnel fixation exhibited an overall complication rate of 30% and those who underwent suture fixation experienced an overall complication rate of 21%. Moreover, 4 patients in the tunnel fixation group exhibited postoperative patellar fracture [11]. An evaluation of five-year outcome of MPFL reconstruction by the basket weave method in a retrospective manner [23] revealed that 4 patients (10.81%) exhibited a positive apprehension test and the mean Kujala anterior knee pain score was measured at 85.15, with the mean Lysholm score reaching 95.30. In our study, however, there were no instances of a positive patellar apprehension test. The mean Kujala knee pain score was 82.79, and the mean Tegner–Lysholm knee score was 82.68.

Efforts have been made globally to develop a standard surgical approach for MPFL reconstruction that minimizes the risk of these complications. This research is subject to specific constraints, including its retrospective nature, the nonrandom selection of participants, a comparatively modest sample size, and a follow-up period limited to only 30 months. A more extensive follow-up spanning up to 5 years would provide more comprehensive insights.

## 5. Conclusion

This surgical technique presents a significant achievement rate, accompanied by the mean Tegner–Lysholm knee score of 82.68 and the mean Kujala anterior knee pain score of 82.71. Notably, there were no complications observed in the postoperative period.

## Data Availability

The data are available at DOI: 10.5061/dryad.9w0vt4bmq.
